# A polyploid admixed origin of beer yeasts derived from European and Asian wine populations

**DOI:** 10.1371/journal.pbio.3000147

**Published:** 2019-03-05

**Authors:** Justin C. Fay, Ping Liu, Giang T. Ong, Maitreya J. Dunham, Gareth A. Cromie, Eric W. Jeffery, Catherine L. Ludlow, Aimée M. Dudley

**Affiliations:** 1 Department of Biology, University of Rochester, Rochester, New York, United States of America; 2 Department of Genetics, Washington University, St. Louis, Missouri, United States of America; 3 Department of Genome Sciences, Seattle, Washington, United States of America; 4 Pacific Northwest Research Institute, Seattle, Washington, United States of America; MIT, UNITED STATES

## Abstract

Strains of *Saccharomyces cerevisiae* used to make beer, bread, and wine are genetically and phenotypically distinct from wild populations associated with trees. The origins of these domesticated populations are not always clear; human-associated migration and admixture with wild populations have had a strong impact on *S*. *cerevisiae* population structure. We examined the population genetic history of beer strains and found that ale strains and the *S*. *cerevisiae* portion of allotetraploid lager strains were derived from admixture between populations closely related to European grape wine strains and Asian rice wine strains. Similar to both lager and baking strains, ale strains are polyploid, providing them with a passive means of remaining isolated from other populations and providing us with a living relic of their ancestral hybridization. To reconstruct their polyploid origin, we phased the genomes of two ale strains and found ale haplotypes to both be recombinants between European and Asian alleles and to also contain novel alleles derived from extinct or as yet uncharacterized populations. We conclude that modern beer strains are the product of a historical melting pot of fermentation technology.

## Abstract 2

Brewer's yeast has been used to make fermented beverages for millennia. A genome analysis of beer and other yeast strains shows that beer yeast have a polyploid admixed origin derived from European and Asian wine populations.

## Author summary

The budding yeast *Saccharomyces cerevisiae* has long been used to make beer. Yeast strains used to make ales are known to differ genetically and phenotypically from strains used to make wine and from strains isolated from nature, such as oak isolates. Beer strains are also known to be polyploid, having more than two copies of their genome per cell. To determine the ancestry of beer strains, we compared the genomes of beer strains with the genomes of a large collection of strains isolated from diverse sources and geographic locations. We found ale, baking, and the *S*. *cerevisiae* portion of lager strains to have ancestry that is a mixture of European grape wine strains and Asian rice wine strains and that they carry novel alleles from an extinct or uncharacterized population. The mixed ancestry of beer strains has been maintained in a polyploid state, which provided a means of strain diversification through gain or loss of genetic variation within a strain but also a means of maintaining brewing characteristics by reducing or eliminating genetic exchange with other strains. Our results show that ale strains emerged from a mixture of previously used fermentation technology.

## Introduction

The brewer's yeast *Saccharomyces cerevisiae* is known for its strong fermentative characteristics. The preference for fermentation in the presence of oxygen arose as a multistep evolutionary process around the time of an ancient genome duplication, endowing numerous species with the ability to produce levels of ethanol toxic to many microorganisms [1,2]. One of these species, *S*. *cerevisiae*, also gained the ability to competitively dominate many other species in high-sugar, low-nutrient environments, such as grape must [3]. Wine is largely fermented by *S*. *cerevisiae* and is thought to be the first fermented beverage, having been made for over 9,000 years [4]. However, *S*. *cerevisiae* is not the only *Saccharomyces* species used to make fermented beverages; others, particularly *S*. *uvarum*, *S*. *kudriavzevii*, *S*. *eubayanus*, and hybrid derivatives, are also used, particularly for fermentations at low temperatures [5–8]. Besides *S*. *cerevisiae*, the most widely used species is *S*. *pastorianus*, an allopolyploid hybrid of *S*. *cerevisiae* and *S*. *eubayanus*, used to make lager beer [7]. The use of this hybrid emerged during the 15th century in Europe and was formed from an *S*. *eubayanus* strain closely related to wild populations from North America and Tibet [9,10] and a *S*. *cerevisiae* strain related to those used to ferment ales [11–13]. The origin of ale and other domesticated strains of *S*. *cerevisiae* is beginning to emerge through comparison with wild populations [12–16].

Multiple genetically distinct populations of *S*. *cerevisiae* have been found associated with fermented foods and beverage. These include grape wine, Champagne, sake and rice wine, palm wine, coffee, cacao, cheese, and leavened bread [14,17–20]. Ale strains have also been found to be both genetically and phenotypically differentiated from other strains [12,13]. Multiple populations of ale strains have been identified and found to exhibit high rates of heterozygosity and polyploidy [12,13,16]. However, the origin of such domesticated groups is not always clear because it requires comparison to wild populations from which they were derived, and these wild populations have not all been identified. The best characterized wild populations of *S*. *cerevisiae* have been isolated from oak and other trees in North America, Japan, China, and Europe [21–24], the latter of which is most closely related to and the presumed wild lineage from which European wine strains were derived.

Despite clear differences among many domesticated groups, human-associated admixture is common [20,22,25,26] and can blur the provenance of domesticated strains. For example, wine strains show a clear signature of admixture with other populations, and clinical strains appear to be primarily derived from admixed wine populations [27–29]. Ale strains, with the exception of a few found related to sake and European wine lineages, have no obvious wild population from which they were derived [12,13].

In this study, we examined the origin of ale and lager strains in relation to a diverse collection of *S*. *cerevisiae* strains. Through analysis of publicly available genomes and 107 newly sequenced genomes, we inferred a hybrid, polyploid origin of beer strains derived from admixture between close relatives of European and Asian wine strains. This admixture suggests that early industrial strains spread with brewing technology to give rise to modern beer strains, similar to the spread of domesticated plant species with agriculture.

## Results

We sequenced the genomes of 47 brewing and baking strains and 65 other strains of diverse origin for reference. Combining these with 430 publicly available genomes, we found nearly all the brewing strains closely related to previously sequenced ale and lager strains (S1 Fig). Through analysis of population structure, we identified 13 populations, 4 of which contain the majority (64/76) of beer strains. The four beer-associated populations consisted of predominantly lager strains, German ale strains (Ale 1), British ale strains (Ale 2), and a mixture of beer and baking strains (Beer/baking) and are consistent with previously identified groups of beer strains [12,13]. The remaining populations were similar to previously characterized groups [20,21,27] and were classified by the most common source and/or geographic region of isolation as Laboratory, Clinical, Asia/sake, Europe/wine, Mediterranean/oak, Africa/Philippines, China/Malaysia, and two populations from Japan/North America (S2 Table).

**Fig 1:**
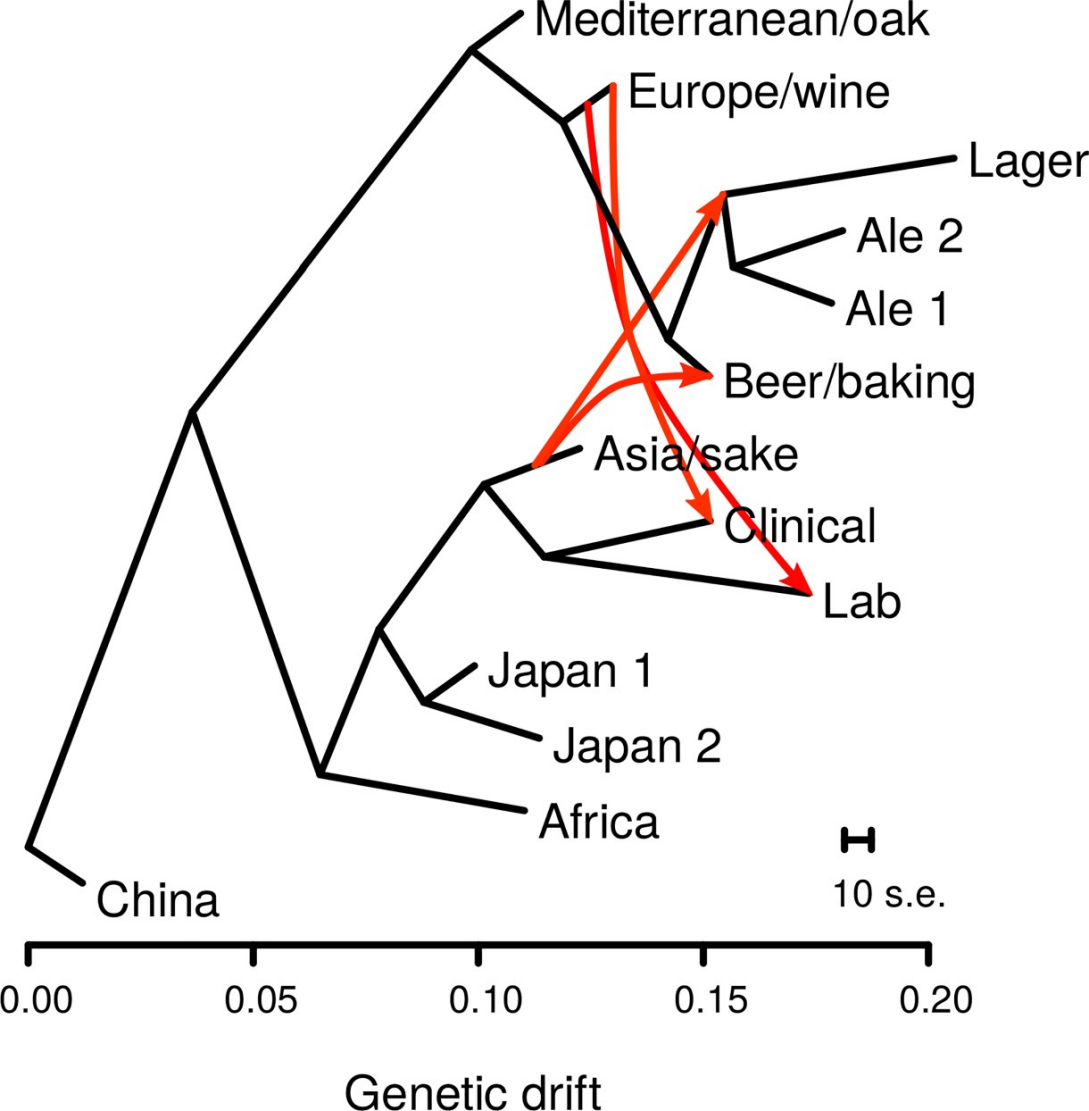
Admixture graph of population relationships shows admixture from the Asia/sake to multiple beer populations. Population relationships were inferred using TreeMix, and horizontal branch lengths are proportional to genetic drift with the scale bar showing the average of 10 s.e. of the sample covariance matrix. Red arrows show admixture events with migration weights over 0.40, indicating the fraction of alleles derived from a source population. Migration from the ancestor of the Mediterranean/oak to the Clinical population is not shown for clarity. The data underlying this figure are available from http://doi.org/10.6084/m9.figshare.7550009.v1. s.e., standard errors.

To identify the most likely founders of the four beer populations, we used a composite likelihood approach to infer population relationships while accounting for admixture, which can obfuscate population phylogenies [30]. The inferred admixture graph grouped the four beer populations together, with the lager and two ale populations being derived from the lineage leading to the Beer/baking population (Fig 1). The four beer populations are most closely related to the Europe/wine population. However, the admixture graph also showed strong support for two episodes of gene flow into the beer lineages resulting in 40% to 42% admixture with the Asia/sake population. We confirmed these admixture events using *f*_*4*_ tests for discordant population trees, which are caused by admixture [31,32]. All *f*_*4*_(Europe, test; Asia, Africa) statistics were significant for tests of the four beer populations (Z-scores < −21.3), whereas the *f*_*4*_(Europe, Mediterranean; Asia, Africa) statistic was much closer to zero for the Mediterranean/oak population (Z-score = 3.4). Similar results were obtained using the China or Japan/North American populations rather than Africa (S3 Table). Therefore, the beer populations were derived from admixture events between a population closely related to the Europe/wine population and Asia/sake population. Consistent with prior studies [20,29], we also found admixture events between the Europe/wine population and both the lab and clinical population.

To quantify the degree of admixture for each of the beer strains, we calculated *f*_*4*_ admixture proportions using the Europe/wine and Asia/sake populations [31,33]. For the 64 beer strains, we estimated an average of 39.6% (range 36.7%–46.7%) of their genome was derived from the Asia/sake population and 60.4% was derived from the Europe/wine population. The high proportion yet narrow range of admixture implies little to no subsequent back-crossing following admixture.

**Fig 2:**
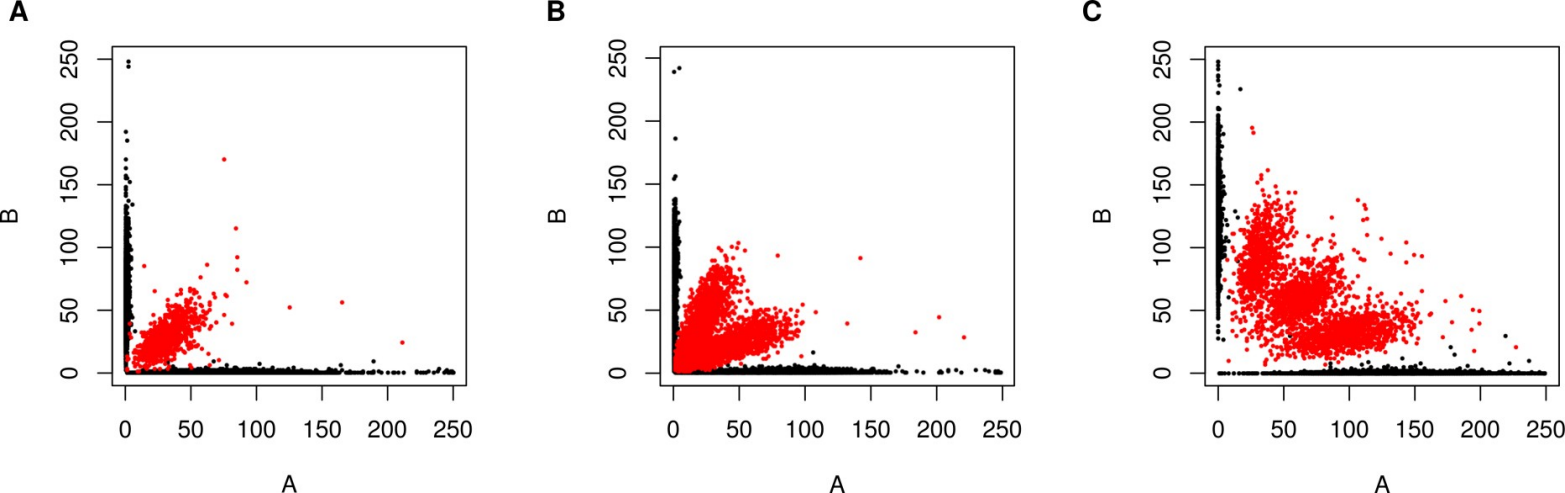
Diploid, triploid, and tetraploids are distinguished by read counts at heterozygous sites. Plots show examples of a (A) diploid (YO700), (B) triploid (TUM205), and (C) tetraploid (YMD1952) strain by the number of reads with the reference allele (A) versus the alternative allele (B) for heterozygous (red) and homozygous (black) sites. The data underlying this figure are available from http://doi.org/10.6084/m9.figshare.7550009.v1.

Polyploidy is enriched in beer and baking strains [12,16,18] and has been shown to contribute to reproductive isolation [34]. The Beer/baking population includes 10 previously studied strains, all of which were found to be triploid or tetraploid and to exhibit high rates of heterozygosity [20]. These strains were previously found to group with other strains isolated from diverse sources around the world (Pan/Mixed 2 in [20]). To identify triploids and tetraploids strains, we used the expected allele frequency at heterozygous sites: 50% for diploids, 33% and 66% for triploids, and 25%, 50%, and 75% for tetraploids (Fig 2). We note that this approach can miss triploid or tetraploid strains due to low heterozygosity or read coverage but should not yield any false positives. Nevertheless, out of 105 strains with an abundance of heterozygous sites, we identified 23 triploid and 28 tetraploid strains (S2 Fig). Of the 51 polyploid strains (N > 2), 45 (88%) were in one of the four beer populations, of which 29 were beer strains and 6 were baking strains. The remaining 10 polyploids assigned to the beer populations include three isolates from green coffee beans and were previously assigned to a Pan/Mixed 2 population [20], a group of predominantly human-associated strains. These results are comparable to the high rates of polyploidy found in prior studies of beer strains [12,16].

**Fig 3:**
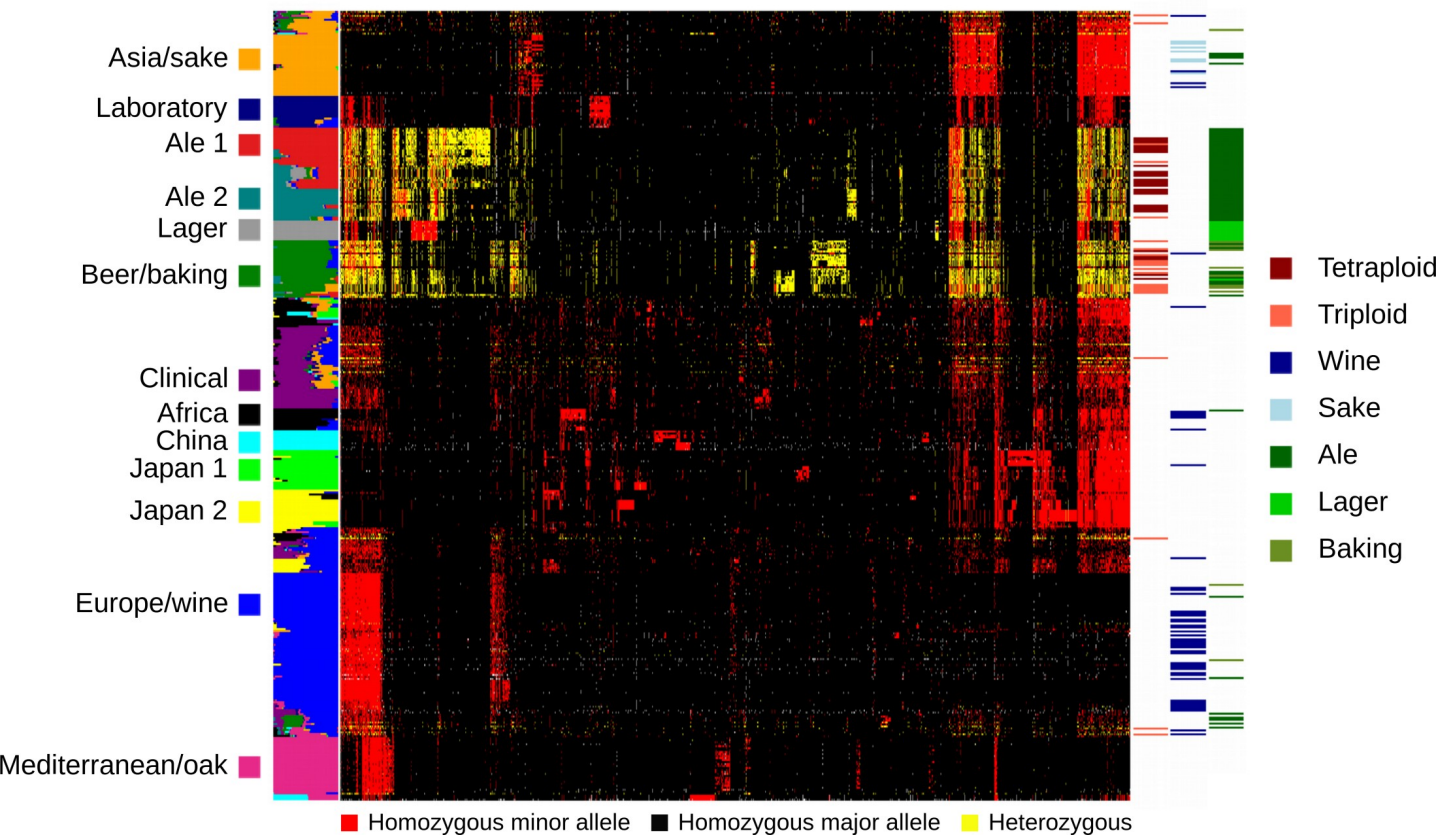
Beer populations are heterozygous for alleles shared with Europe/wine and Asia/sake populations. Genotypes of 2,000 randomly selected SNPs are shown for 339 strains grouped by their ancestry to 13 populations shown by the colored panel on the left. Genotypes are homozygous for the major allele (black), minor allele (red), or heterozygous (yellow), and SNPs (columns) were ordered by hierarchical clustering. The panel on the right indicates triploid and tetraploid strains, grape and sake wine strains, and ale, lager, and baking strains. The data underlying this figure are available from http://doi.org/10.6084/m9.figshare.7550009.v1.

Given the admixed origin of beer strains, we wanted to know which populations the heterozygous sites indicative of polyploidy were derived from. We examined heterozygous sites in the beer populations in relation to other strains by clustering SNPs and grouping strains by their inferred population membership (Fig 3). Excluding the lager population, which are not heterozygous, the three beer populations are predominantly heterozygous for alleles abundant within either the Asia/sake or Europe/wine population. However, all four beer populations also carry alleles not present in any of the other populations. The presence of heterozygous, beer-specific alleles suggests that these alleles were derived from admixture between a lineage closely related to the Europe/wine lineage and/or the Asia/sake lineage. The large number of beer-specific alleles is unlikely to have accumulated in the recent past subsequent to the formation of the polyploids. The beer groups have between 6,558 and 13,728 alleles present at 25% frequency or more in the group but not in any other population. Using these beer-specific alleles, we found the divergence at four-fold degenerate synonymous sites was 0.153%, 0.100%, 0.087%, and 0.069% along the Ale 1, Ale 2, Beer/baking, and Lager lineages. These rates are higher than expected to have accumulated since the use of these strains for brewing purposes (see Discussion) and not much less than the rate of divergence between the Europe/wine and Asia/sake population (0.592%). Combined with the observation that these variants are mostly heterozygous, we infer that many were present when the strains became polyploid and originated from an extinct or as yet to be characterized yeast population related to the Europe/wine and/or Asia/sake population.

The presence of heterozygous European, Asian, and beer-specific alleles enabled us to use haplotype phasing to test whether there has been recombination between European and Asian haplotypes and whether beer-specific alleles reside on European or Asian haplotypes. We used long-read sequencing to phase two beer strains—a German ale strain in the Ale 2 population (A.2565) and a Belgian ale strain in the Beer/baking population (T.58). Both strains were inferred to have over 99% ancestry to their assigned population, and both are likely polyploids (S2 Table). As a control, we phased the genome of a hybrid (YJF1460) generated between a Europe/wine strain and a Japan/North America 2 oak strain. Because of uncertainty in the ploidy as well as the possibility of variable ploidy levels (aneuploidy) across the genome, we developed a phasing algorithm that merges reads into consistent haplotypes and makes no assumptions about ploidy.

**Fig 4:**
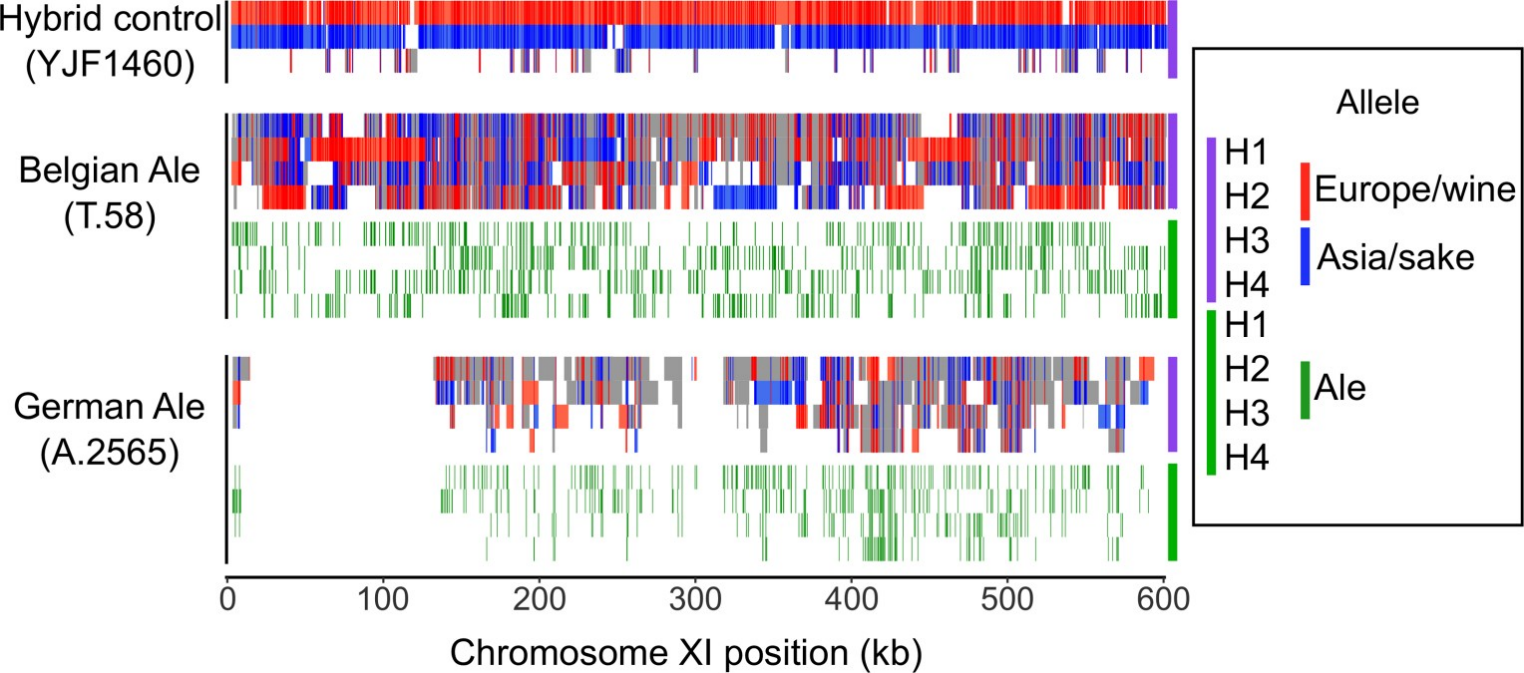
Phased haplotypes show recombination between European and Asian alleles. Panels for two ale strains (T.58 and A.2565) and the control hybrid (YJF1460) show haplotypes and allele configurations across chromosome XI. The purple panel shows haplotypes with more than 95% European or Asian alleles in red and blue, respectively, and in gray otherwise. Ticks indicate European (red) and Asian (blue) alleles. Haplotypes were assigned labels H1–H4 in order of longest to shortest, except for YJF1460 in which they were assigned based on predominance of Europe/wine (H1, red) or Asia/sake (H2, blue) alleles. The green panel shows ale-specific alleles for the four haplotypes (H1–H4) by green ticks. The first 100 kb of A.2565 shows a region exhibiting loss of heterozygosity. The data underlying this figure are available from http://doi.org/10.6084/m9.figshare.7550009.v1.

Phasing of the three strains yielded predominantly two haplotypes in the YJF1460 control and three or four haplotypes in the two ale strains (Fig 4 and S4 Fig). The majority of phased haplotypes in the two ale strains carried a mixture of European and Asian alleles. In contrast, the YJF1460 control showed few haplotypes with both European and Asian alleles, which is indicative of haplotype switching errors or mitotic exchange. Eliminating regions of haplotype switching less than 4 kb in length, which could result from genotype errors or mitotic gene conversion, we counted the number of switches within the phased haplotypes between European and Asian alleles and found 12 in YJF1460, 346 in T.58, and 199 in A.2565 (S4 Table). Consequently, most haplotypes present in the two ale strains represent recombinant haplotypes as opposed to pure European-related or Asian-related haplotypes (Fig 4 and S4 Fig); only 22% of the T.58 genome and 19% of the A.2565 genome carried haplotypes with over 95% European or 95% Asian alleles. In contrast, 88% of the YJF1460 genome was inferred to carry pure European or Asian haplotypes.

The amount of recombination between European and Asian alleles is indicative of time since admixture. We measured the decay in linkage disequilibrium between European and Asian alleles on phased haplotypes as a function of distance and found a 50% drop in linkage disequilibrium corresponds to 6.3 kb in A.2565 and 30 kb in T.58 and no decay in YJF1460 (S5 Fig). Assuming 0.34 kb/cM [35], this translates to an equivalent of 46.9 meiotic events in A.2565 and 9.8 meiotic events in T.58. For comparison, we estimated the number of meiotic equivalents since admixture of the Clinical (9.5), Laboratory (13.7), Lager (72.1), Beer/baking (6.7), Ale 1 (75.0), and Ale 2 (67.5) populations. These results indicate more recent admixture of the Clinical, Laboratory, and Beer/Baking populations compared with the Lager and two ale populations.

Although it is difficult to know how much recombination occurred prior to polyploidy and how much occurred subsequently through mitotic recombination or gene conversion, mitotic events have contributed to diversification of beer strains subsequent to polyploidy. There are four large and a number of smaller regions in A.2565 that exhibit loss of heterozygosity (Fig 4), and loss of heterozygosity is a distinct signature of mitotic recombination. Recombination of European- and Asian-derived haplotypes occurred prior to loss of heterozygosity because the fixed haplotypes in regions where there is loss of heterozygosity are recombinants of European- and Asian-derived haplotypes.

The polyploid beer strains also carry beer-specific alleles not present in other strains. These beer-specific alleles could have been inherited from an ancestral population that split from either the European/wine or Asian/sake population, or from an admixed population. To distinguish between these possibilities, we examined the distribution of beer-specific alleles on the phased haplotypes and found most (approximately 80%) were on mixed haplotypes, having at least 5% European and 5% Asian alleles. Therefore, beer-specific alleles were present during admixture of European and Asian alleles. The remaining beer-specific alleles were equally distributed between predominantly European or predominantly Asian haplotypes (S4 Table). We also counted alleles at heterozygous sites as an indicator of ploidy contribution. At sites with four phased haplotypes, we found Asian alleles had an average of 1.93 and 1.96 copies in T.58 and A.2565, respectively. In contrast, beer-specific alleles had an average 1.31 and 1.56 copies in T.58 and A.2565, respectively. The lower copy number of beer-specific alleles suggests either lower beer-specific allele frequencies compared to European/Asian allele frequencies in the ancestral admixed population prior to polyploidy or that polyploidy involved parents from populations with unequal representation of beer-specific alleles compared to European/Asian alleles. Finally, because many of the beer-specific alleles are not shared between the Ale 1, Ale 2, Beer/baking, and Lager populations, we can infer multiple origins of the four beer populations despite similar episodes of admixture and polyploidy, demonstrating that the *S*. *cerevisiae* contribution to lager strains did not simply come from one of the other beer strain lineages.

## Discussion

Inferring the origin of domesticated organisms can be complicated by extinction of wild progenitor populations, human-associated migration, polyploidy, and admixture with wild populations. In this study, we find that extant beer strains are polyploid and have an admixed origin between close relatives of European and Asian wine strains. Ale genomes, like lager genomes, carry relics of their parental genomes captured in a polyploid state as well as novel beer alleles from an extinct or undiscovered population. Loss of heterozygosity through mitotic exchange provided a means of strain diversification but has also potentially eroded precise inference of the timing and order of events giving rise to modern beer strains. Below, we discuss models and implications for an admixed, polyploid origin of beer strains.

Polyploidy is thought to mediate rapid evolution [36], and prior work showed that polyploidy is common in beer and baking strains [12,18,31]. We find that the Ale 1, Ale 2, and Beer/baking population all have a polyploid origin. Although not all strains had sufficient coverage for calling polyploidy, all those that did were either triploid or tetraploid. Chromosome level aneuploidy is also more common in strains within the Ale 1 (52%), Ale 2 (19%), and Beer/baking (52%) populations than in the nonbeer populations (5.1%). A notable consequence of both polyploidy and aneuploidy is that they can limit admixture with haploid or diploid strains due to low spore viability [34,37,38], thereby maintaining their brewing characteristics. Indeed, beer strains exhibit low sporulation efficiency and spore viability [12]. Both grape wine and particularly sake wine strains have also evolved more limited capacities to interbreed through low sporulation efficiencies [39,40].

Human-associated admixture is well documented in wine strains, which have been dispersed around the globe with the spread of viticulture [20,22,25,26]. However, admixture between close relatives of European grape wine and Asian rice wine populations presents a conundrum regarding where and how these populations became admixed. A crucial yet unresolved piece of information is where European wine strains were domesticated. The discovery of a Mediterranean oak population closely related to European wine strains suggests a European origin of wine strains [21]. An alternative model is that the Mediterranean oak population is a feral wine population and both the European wine and Mediterranean oak populations are nonnative. Analysis of a diverse collection of Asian strains suggested an East Asian origin of all domesticated *S*. *cerevisiae* strains, including European wine strains [14]. Domestic populations from solid and liquid state fermentations (bread, milk, distilled liquors, rice wines, and barley wines) were found related to wild populations from East Asia. In support of European wine and Mediterranean oak populations also originating in East Asia, these populations carry duplicated genes involved in maltose metabolism and grouped with fermented milk and other strains isolated from China. However, this model also has some uncertainty given the small number of Chinese isolates within the European wine group, the dispersion of European wine strains with viticulture, and the absence of samples from the Caucasus where grapes are thought to have been domesticated [4,41].

Considering the uncertainty of where European wine strains were domesticated, we put forth two hypotheses regarding the admixed origin of beer strains. First, European wine strains were domesticated in East Asia and admixed in situ with a population related to the Asia/sake group, which contains eight sake/rice wine strains, seven distillery strains, and seven bioethanol strains, mostly from Asia. Second, European wine strains were domesticated in Europe from a Mediterranean oak population, or perhaps in the Caucasus, and the admixed beer populations arose through East–West transfer of fermentation technology, including yeast by way of the Silk Route. Resolving these scenarios would be greatly facilitated by finding putative parental populations of diploid but not necessarily wild strains that carry alleles we find to be unique to the Ale 1, Ale 2, Beer/baking, and Lager groups. As yet, such populations have not been sampled or are extinct.

Even with a clear signature of a polyploid and admixed origin of beer strains, there are uncertainties regarding the founding strains and the order of events. The decay in linkage disequilibrium suggests that admixture occurred prior to polyploidy, and the distribution of beer-specific alleles suggests that admixture involved at least one uncharacterized population. However, polyploid genomes are often labile, and it is hard to know the extent to which mitotic recombination and gene conversion have altered genetic variation in the beer strains. In yeast, the rate of mitotic gene conversion and recombination has been estimated to be 1.3 × 10^−6^ per cell division and 7 × 10^−6^ per 120 kb, respectively [42,43], and both can lead to loss of heterozygosity. Converting to the size of a tetraploid genome (approximately 48 Mbp), we expect 0.0038 (using a median track length of 16.6 kb) conversion events and 0.0028 recombination events across the genome per cell division. Three lines of evidence support the role of these mitotic events in beer strains. First, many of the switches between the European and Asian alleles involved one or a small number of adjacent SNPs rather than long segments, indicative of gene conversion (S4 Table). Second, one strain (A.2565) shows clear loss of heterozygosity on multiple chromosomes, indicative of mitotic recombination (S4 Fig). Third, there is substantial genotype diversity within each of the beer populations (Fig 3). This would be expected to occur if loss of heterozygosity occurred during strain divergence but subsequent to the founding of each beer population.

Two other factors besides mitotic gene conversion and recombination must be considered in regards to diversity within the beer populations—outcrossing and de novo mutation. Outcrossing with strains outside of the beer population is unlikely because there is no evidence for this type of admixture in our analysis and admixture proportions from the Asian population is fairly constant at 37% to 47% across beer strains. However, it is worth noting that outcrossing of strains within or between different beer populations may not easily be detected. De novo mutations have undoubtedly occurred, but even using a reasonable estimate of 150 generations per year for brewing strains [12] and a per base mutation rate of 5 × 10^−10^ [44], the beer lineage substitution rates yield divergence times of 2.0 × 10^4^ (Ale 1), 1.3 × 10^4^ (Ale 2), 1.1 × 10^4^ (Beer/baking), and 9.2 × 10^3^ (Lager) years. Therefore, a sizable fraction of beer-specific alleles was likely inherited from populations closely related to European wine and Asian wine populations rather than de novo mutations that accumulated subsequent to polyploidy. Regardless of the relative impact of mitotic recombination, gene conversion, outcrossing, and de novo mutation, beer strains have diversified from one another but have remained relatively distinct from other populations of *S*. *cerevisiae* [12,13].

In conclusion, beer strains are the polyploid descendants of strains related to but not identical to European grape wine and Asian rice wine strains. Therefore, similar to the multiple origins of domesticated plants, including barley [45] and rice [46,47], beer yeasts are the products of admixture between different domesticated populations and benefited from historical transfer of fermentation technology.

## Materials and methods

### Genome sequencing and reference genomes

Genome sequencing was completed for 47 commercial yeast strains, which include 33 ale, 7 lager, 2 whiskey, and 5 baking strains. For reference, sequencing was also completed for 60 strains of diverse origin, including 22 isolates from trees or other nonhuman-associated sources and 38 isolates from human-associated ferments such as togwa, coffee, and cacao (S1 Table). For each strain, DNA was extracted and indexed libraries were sequenced on Illumina machines (NextSeq, HiSeq2000, or HiSeq2500). A median of 10.7 million reads per strain was obtained, ranging from 272,000 to 26 million. The sequencing data is available at NCBI (PRJNA504476).

Genomic data was obtained for 430 strains from publicly available databases. These include 138 additional beer strains from [12,13]. We also obtained reference genomes for *S*. *paradoxus*, *S*. *mikatae* [48], and *S*. *eubayanus* (SEUB3.0) [49]. Two large sets of recently published genomes [14,16] were obtained for comparison with our set of 537 genomes. Genotype calls for SNPs identified in this study were obtained from gvcf files of the 1,011 yeast genomes project [16], and genotype calls were generated for 266 strains from China [14] using the mapping and genotyping pipeline described below. Because these two later sets of data were only available recently, they were only incorporated into the S1 Fig heatmap.

### Alignment, variant calling, and genotyping

Reads were aligned to the *S*. *cerevisiae* S288c reference genome (R64-1-1_20110203) using BWA-v0.7.12-r1039 [50]. Lager strains were mapped to a concatenated *S*. *cerevisiae* and *S*. *eubayanus* genome and reads mapping to *S*. *eubayanus* were discarded. For short reads (<70 bp), we used BWA-sampe, and for the remainder, we used BWA-mem. Duplicate reads were marked prior to genotyping. Assembled genomes were also mapped using BWA-mem, and flags for secondary alignments were removed to facilitate complete mapping of large contigs. For *S*. *paradoxus* and *S*. *mikatae*, we obtained higher coverage of the S288c genome by mapping synthetic reads fromshredded contigs compared to mapping of full contigs and so used the former.

SNPs were called using short read data and then genotyped in those strains with assembled genomes. For SNP calling, we used GATK-UnifiedGenotyper-v3.3–0 [51] and applied the hard filters: QD < 5, FS > 60, MQ < 40, MQRankSum < −12.5, and ReadPosRankSum < −8. The dataset was filtered to remove strains and sites with more than 10% missing data. Among those strains removed were lager strains of the type 1 Saaz group [11], but we retained *S*. *paradoxus* and *S*. *mikatae* for which we obtained calls at 78% and 40% of sites, respectively. Biallelic SNPs with a minor allele frequency of at least 1% and with at least four minor allele genotype calls were selected for analysis, resulting in a total of 273,963 SNPs. The 399 strains retained for analysis are listed in S2 Table, and the genotype data is available in variant call format from http://doi.org/10.6084/m9.figshare.7550009.v1. Genotype calls for these SNPs were also obtained for the 1,277 strains in the comparative data set [14,16].

To estimate our genotyping error rate, we compared six pairs of strains that were independently sequenced. Two of the strains, YJF153 and BC217, were haploid derivatives of diploids strains, YPS163 [52] and BC187 [53], respectively, that were also sequenced. The other four pairs were all beer strains independently obtain from Wyeast (Wyeast 1728, 1968, 2565, 2112) and independently sequenced at Washington University in St. Louis and University of Washington in Seattle. Between the pairs of strains, we found genotype discordance rates of 9.62 × 10^−4^ (YJF153/YPS163), 1.31 × 10^−3^ (BC217/BC187), 3.57 × 10^−3^ (L.2112/YMD1874), 3.00 × 10^−3^ (A.2565/YMD1952), 1.81 × 10^−2^ (A.1968/YMD1981), and 5.74 × 10^−3^ (A.1728/YMD1866). We retained the six pairs of strains throughout the analysis as a measure of robustness.

### Ploidy and aneuploidy

Ploidy and aneuploidy were assessed by read counts at heterozygous sites and read coverage, respectively. For ploidy analysis, genotypes of 317 strains were from assemblies, and so no information on heterozygous sites was available, and 117 strains had few heterozygous sites indicating they were haploid or homozygous diploid. Of the remaining 105 strains, 66 had sufficient coverage at heterozygous sites to make visual designations of ploidy [20,54,55]. Visual designations were based on dominant trends consistent with expected percentage of read counts supporting—diploid (50:50), triploid (33:66), tetraploid (25:50:75) allele configurations. Of the 39 strains without sufficient coverage to distinguish triploids from tetraploids, most (33) showed distributions consistent with polyploidy (ploidy > 2), and of these, 29 were beer strains (S2 Fig). Aneuploidy was assessed by visual inspection of read coverage across the genome. Aneuploidy was only called for clear cases in which one or more chromosomes showed a deviation in read coverage compared to all other chromosomes.

### Population structure and admixture

Population structure was inferred by running ADMIXTURE [56] on a set of 20,394 sites with a minimum physical distance of 500 bp. The variants from 138 strains in a recent study of beer strains [12] were removed because the assemblies eliminated heterozygous sites and raw reads for these genomes were not available. Based on 20 independent runs using between 4 and 20 populations for the 399 strains, we chose 13 based on an average change in the log-likelihood greater than 3 standard deviations of the variation in the log-likelihood among independent runs (S3 Fig). The beer populations of interest were not affected by this choice; with 12 populations, the 2 Japanese populations merged and with 14—a new population of admixed European wine strains was formed (S3 Fig).

Population admixture graphs were inferred using Treemix [30]. A subset of 199 strains with less than 1% admixture were used to generate a population admixture graph. The population from China was used to root the tree because two strains in the China population, HN6 and SX6, were most closely related to both *S*. *paradoxus* and *S*. *mikatae*, and blocks of 500 SNPs were used to obtain jacknife standard errors. Five episodes of migration were inferred (P < 4.9 × 10^−12^), with weights ranging from 0.18 to 0.49. Migration events were validated using *f*_*4*_ tests of admixture (S3 Table). For tests of tree discordance, we did not use the clinical and lab populations as reference populations because these showed evidence of admixture. *f*_*4*_ admixture proportions were estimated by the ratio of *f*_*4*_(Mediterranean, Africa; test, Europe) to *f*_*4*_(Mediterranean, Africa; Asia, Europe), in which each of the 64 beer strains in the Ale 1, Ale 2, lager, and beer/baking populations were individually tested.

### Long-read phasing

Three strains were selected for PacBio sequencing and variant phasing. Two of the strains were beer strains, A.2565 and A.T58, and the third, YJF1460, was a hybrid we generated by mating a European/wine strain (BC217) and a Japan/North America 2 oak strain (YJF153). PacBio reads were aligned to the S288c reference genome using Blasr [57], and heterozygous variants in each genome were phased using HapCUT2 [58], and our own heuristic phasing method that accounts for variable ploidy levels across the genome. Average coverage at 56k, 59k, and 33k variant sites was 13.1, 18.8, and 13.0 for YJF1460, A.T58, and A.2565, respectively. Our custom phasing method used the variant call format files and fragment files from HapCUT2 as input, and output a variable number of phased haplotypes. HapCUT2 fragment files were generated with minimum base quality of 10. Reads were merged into haplotypes using a minimum overlap of four matching SNPs and a minimum of 80% matching SNPs. Reads were iteratively joined to haplotypes using the best scoring overlap based on score = matches– 5 × mismatches. Haplotypes were formed by three rounds of merging. In the first round, reads were merged into haplotypes without any mismatches. In the second and third rounds, haplotypes were merged using the criteria defined above. Error rates were estimated by counting the minimum number of mismatches of reads to the final set of haplotypes. Error rates of 1.84%, 2.03%, and 1.90% were obtained from comparison of reads to 3337, 2452, and 2607 haplotype alleles for YFJ1460, A.T58, and A.2565, respectively. The average number of haplotypes at phased sites was 2.29, 3.27, and 2.98 for YFJ1460, A.T58, and A.2565, respectively. Sites where three haplotypes were inferred in the YJF1460 control are largely due to overlapping haplotypes that were too short to merge. The long read data, custom phasing script and inferred haplotypes are available from http://doi.org/10.6084/m9.figshare.7550009.v1.

After phasing, two sets of SNPs were selected for analysis. The first set consisted of nearly fixed differences between the Europe/wine and Asia/sake populations. After excluding strains with more than 1% admixture, there were 34,022 sites with an allele frequency of 99% in Europe/wine strains (*n* = 47) and less than 1% frequency in Asia/sake strains (*n* = 28) or vice versa. The nearly fixed differences between Europe/wine and Asia/sake strains were used to quantify switching between European and Asian haplotypes. Switching events were measured by counting switches involving one or more sites, five or more sites, or sites spanning 4 kb or longer (S4 Table). The latter two measures were used to avoid counting switches caused by sequencing errors or mitotic gene conversion events, which should not affect multiple adjacent sites or regions longer than 4 kb [59], respectively. The switching rate for the YJF1460 control was similar to that obtained using HAPCUT2 (S4 Table), which minimizes errors when merging reads but assumes a ploidy of two, and SDhaP [60] run assuming a ploidy of two for YJF1460 and four for the two ale strains. The second set consisted of alleles abundant in the four beer populations but absent in all others. After excluding strains with more than 1% admixture, there were 32,829 sites with allele frequencies over 25% in either the Ale 1 (n = 13), Ale 2 (n = 12), or Beer/baking strains (n = 2), but less than 1% in all other populations. To avoid problems with low-coverage strains, we estimated population allele frequencies from counts of homozygous calls and half of heterozygous calls.

Decay in linkage disequilibrium was measured by the covariance in alleles between sites [61]. An exponential decay function was fit to the average covariance of sites binned every 100 bp from 1kb to 50kb. Rather than weight linkage disequilibrium based on the allele frequency differences between the two admixed populations, we used the unweighted covariance across 34,022 sites that show nearly fixed differences between the Europe/wine and Asia/sake population. For the phased strains, we used the covariance across sites on the same haplotypes. For the population decay estimates, we only used strains with 99% or more ancestry assigned to either the Clinical, Laboratory, Ale 1, Ale 2, Beer/baking, and Lager populations. Invariant sites were excluded in each case. We assumed 0.34 kb/cM [35] to translate decay in physical distance to genetic distance and infer the number of meiotic equivalents.

We estimated divergence using four-fold degenerate sites in coding sequences. Excluding splice sites and sites with overlapping gene annotations, there were 1,036,317 four-fold degenerate sites surveyed. At these sites, we found 1586, 1040, 899, and 716 alleles at a frequency of 25% or more in the Ale 1, Ale 2, Beer/baking, or Lager population, respectively, but not in any other population.

## Supporting information

S1 Fig Heatmap of clustered genotypes and strains relating other studies to this one. Strains are color coded by bars, from left to right. Column 1: beer strains (red) with grey labels from Peter and colleagues [16] (African Beer, Mosaic Beer) or this study (Ale+Lager); column 2: referenced study; column 3: population assignments from this study. Strains (rows) and SNPs (columns) show genotypes: major allele homozygous (black), heterozygous (yellow), and minor allele homozygous (red). The data underlying this figure are available from http://doi.org/10.6084/m9.figshare.7550009.v1.
(PDF)

S2 Fig Ploidy inferred from the frequency of genotyped SNPs. Each graph shows the frequency of reads with the reference (A) versus the nonreference (B) allele, with color indicating the genotype call (black = homozygous, red = heterozygous). Diploids, triploids, and tetraploids were inferred by heterozygous SNPs being predominantly at frequencies of 50, 33:66, and 25:50:75, respectively. The data underlying this figure are available from http://doi.org/10.6084/m9.figshare.7550009.v1.
(PDF)

S3 Fig Fit of admixture models as a function of the number of populations. (A) Boxplot of the log-likelihood of 20 independent runs as a function of the number of populations. (B) The scaled improvement in fit, measured by the change in the log-likelihood with increasing population number divided by the standard deviation in the log-likelihood values from 20 independent runs. (C) Population assignments assuming a different number of populations. Each row shows a strain with ancestry to different populations shown by colors and population labels based on similarity to the labels for 13 populations. The data underlying this figure is available from http://doi.org/10.6084/m9.figshare.7550009.v1.
(PDF)

S4 Fig Phased haplotypes show recombination between European and Asian alleles. Panels are the same as in Fig 4 and show all 16 chromosomes for two ale strains (T.58 and A.2565) and the control hybrid (YJF1460). European or Asian alleles are shown in red and blue, respectively, and ale alleles in green. The orange panel shows homozygous ale, European and Asian alleles, as well as sites heterozygous for Europe-Asia alleles. The data underlying this figure are available from http://doi.org/10.6084/m9.figshare.7550009.v1.
(PDF)

S5 Fig Decay in linkage disequilibrium as a function of distance between sites. Linkage disequilibrium was measured by the covariance of alleles across sites on the same phased haplotypes for A.2565 (red), T.58 (green), and YJF1460 (blue). Each point shows the average covariance of sites with distances binned into 100 bp increments. The solid lines represent the fit to an exponential decay function. The data underlying this figure are available from http://doi.org/10.6084/m9.figshare.7550009.v1.
(PDF)

S1 Table Strains sequenced in this study. (XLSX)

S2 Table Strain sources, population assignments and ploidy. (XLSX)

S3 Table f4 tests for admixture based on tree discordance. (XLSX)

S4 Table Haplotype switching and beer-specific alleles. (XLSX)
