# Supplementary material for: A polyploid admixed origin of beer yeasts derived from European and Asian wine populations
Source: PLoS Biol. 2019 Mar 5;17(3):e3000147. doi: 10.1371/journal.pbio.3000147 (PMC6400334; doi:10.1371/journal.pbio.3000147)
Supplement: S1 Fig — Strains are color coded by bars, from left to right. Column 1: beer strains (red) with grey labels from Peter and colleagues [16] (African Beer, Mosaic Beer) or this study (Ale+Lager); column 2: referenced study; column 3: population assignments from this study. Strains (rows) and SNPs (columns) show genotypes: major allele homozygous (black), heterozygous (yellow), and minor allele homozygous (red). The data underlying this figure are available from http://doi.org/10.6084/m9.figshare.7550009.v1. (PDF) [file pbio.3000147.s001.pdf]

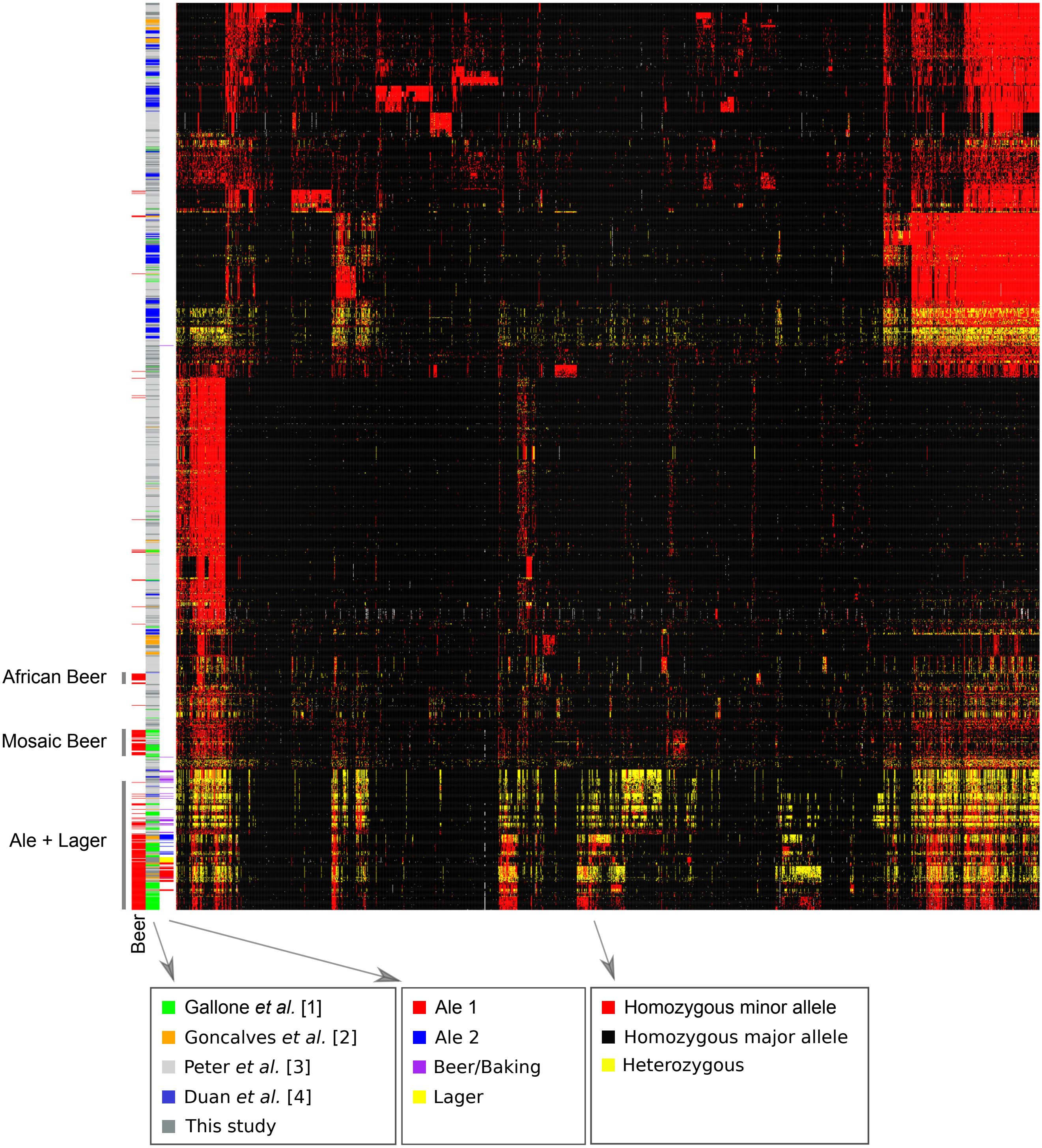

References

[1] Gallone B, Steensels J, Prahl T, Soriaga L, Saels V, Herrera-Malaver B, et al. Domestication and Divergence of Saccharomyces cerevisiae Beer Yeasts. Cell. 2016;166: 1397-1410.e16. doi:10.1016/j.cell.2016.08.020

[2] Gonçalves M, Pontes A, Almeida P, Barbosa R, Serra M, Libkind D, et al. Distinct Domestication Trajectories in Top-Fermenting Beer Yeasts and Wine Yeasts. Curr Biol. 2016;26: 2750–2761. doi:10.1016/j.cub.2016.08.040

[3] Peter J, De Chiara M, Friedrich A, Yue J-X, Pflieger D, Bergström A, et al. Genome evolution across 1,011 Saccharomyces cerevisiae isolates. Nature. 2018;556: 339–344. doi:10.1038/s41586-018-0030-5

[4] Duan S-F, Han P-J, Wang Q-M, Liu W-Q, Shi J-Y, Li K, et al. The origin and adaptive evolution of domesticated populations of yeast from Far East Asia. Nat Commun. 2018;9: 2690. doi:10.1038/s41467-018-05106-7
