# Supplementary material for: A polyploid admixed origin of beer yeasts derived from European and Asian wine populations
Source: PLoS Biol. 2019 Mar 5;17(3):e3000147. doi: 10.1371/journal.pbio.3000147 (PMC6400334; doi:10.1371/journal.pbio.3000147)
Supplement: S2 Fig — Each graph shows the frequency of reads with the reference (A) versus the nonreference (B) allele, with color indicating the genotype call (black = homozygous, red = heterozygous). Diploids, triploids, and tetraploids were inferred by heterozygous SNPs being predominantly at frequencies of 50, 33:66, and 25:50:75, respectively. The data underlying this figure are available from http://doi.org/10.6084/m9.figshare.7550009.v1. (PDF) [file pbio.3000147.s002.pdf]

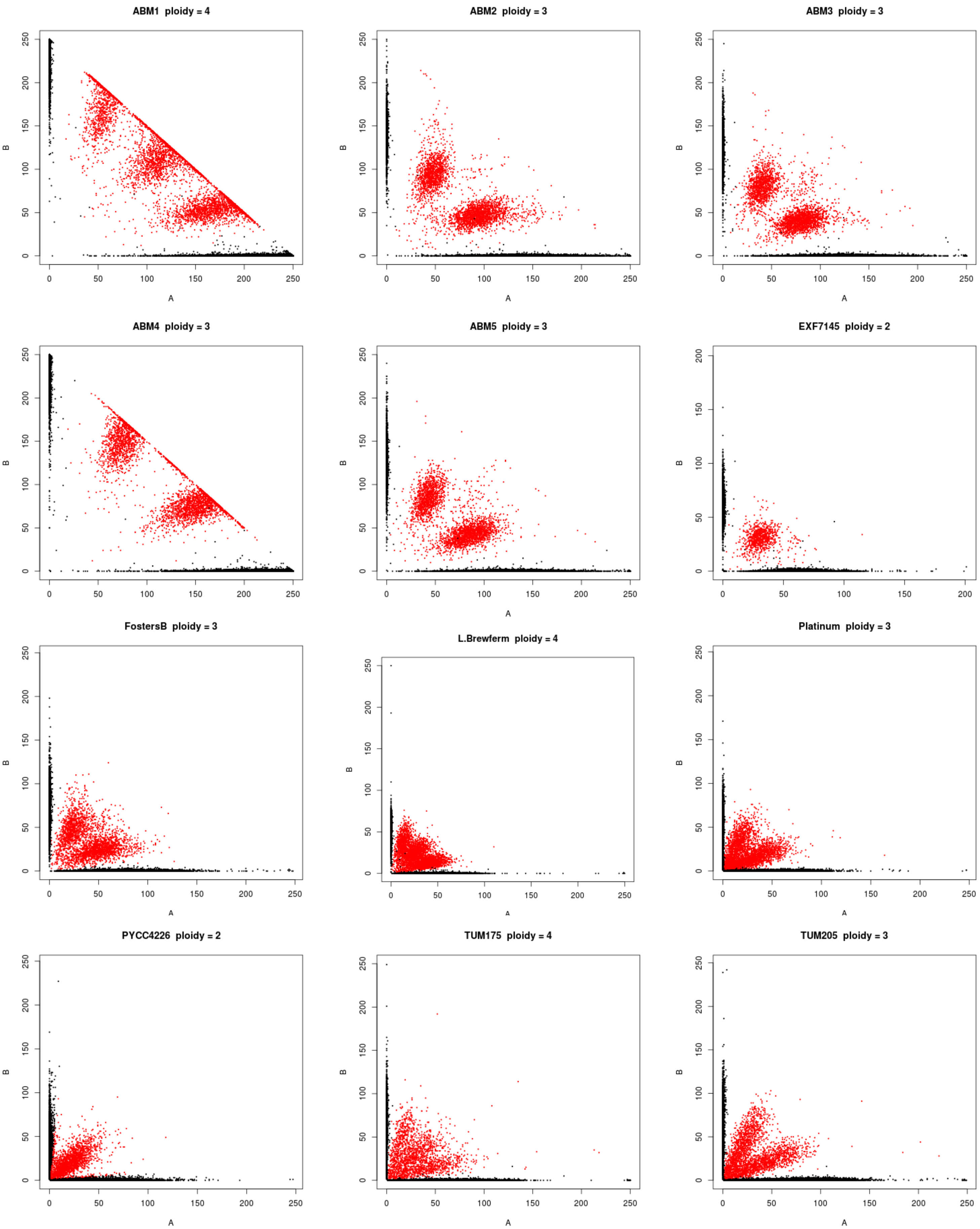
ABM1 ploidy = 4
ABM2 ploidy = 3
ABM3 ploidy = 3
ABM4 ploidy = 3
ABM5 ploidy = 3
EXF7145 ploidy = 2
FostersB ploidy = 3
L.Brewferm ploidy = 4
Platinum ploidy = 3
PYCC4226 ploidy = 2
TUM175 ploidy = 4
TUM205 ploidy = 3
A
B
0
50
100
150
200
250

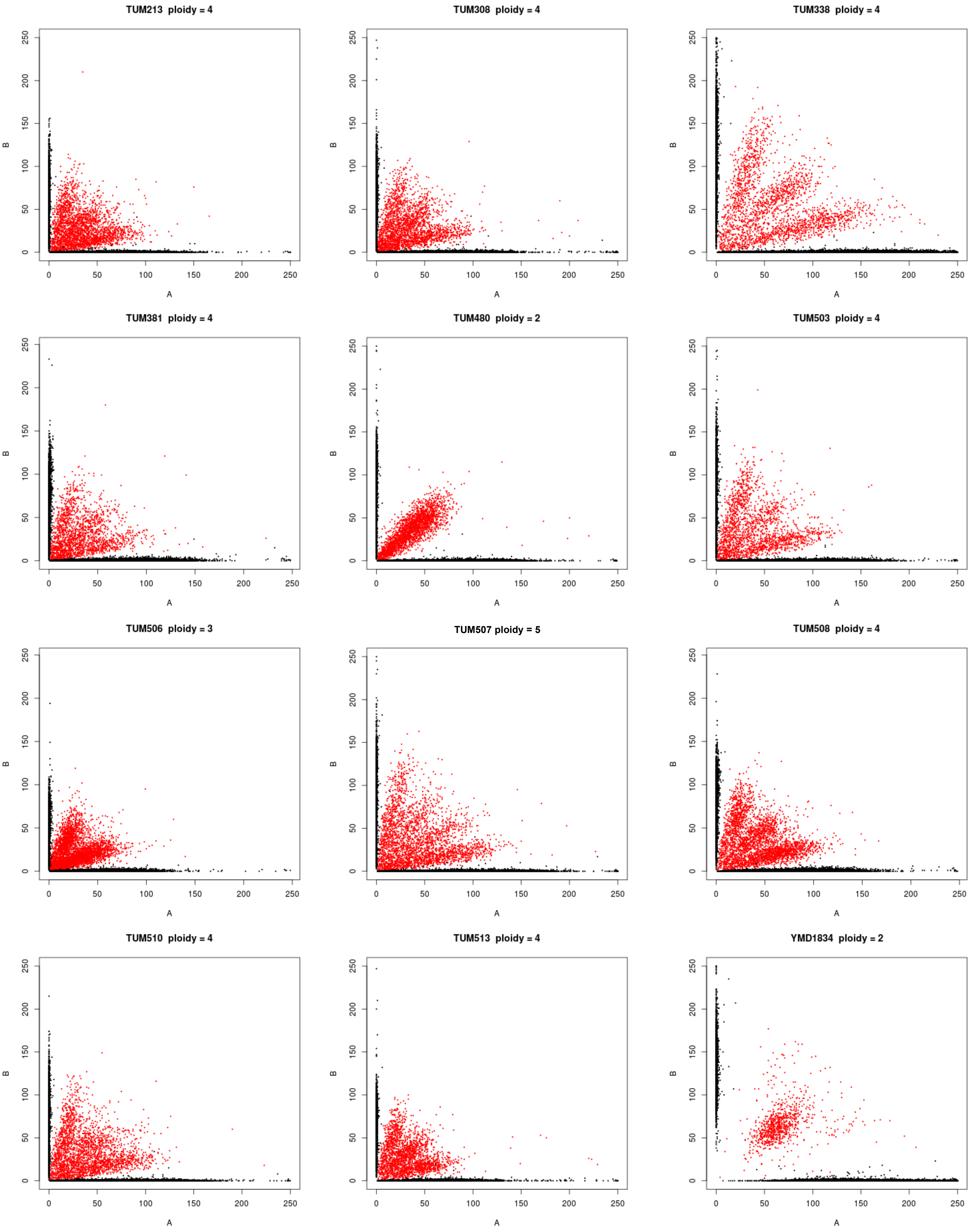
TUM213 ploidy = 4
TUM308 ploidy = 4
TUM338 ploidy = 4
TUM381 ploidy = 4
TUM480 ploidy = 2
TUM503 ploidy = 4
TUM506 ploidy = 3
TUM507 ploidy = 5
TUM508 ploidy = 4
TUM510 ploidy = 4
TUM513 ploidy = 4
YMD1834 ploidy = 2
A
B
0
50
100
150
200
250

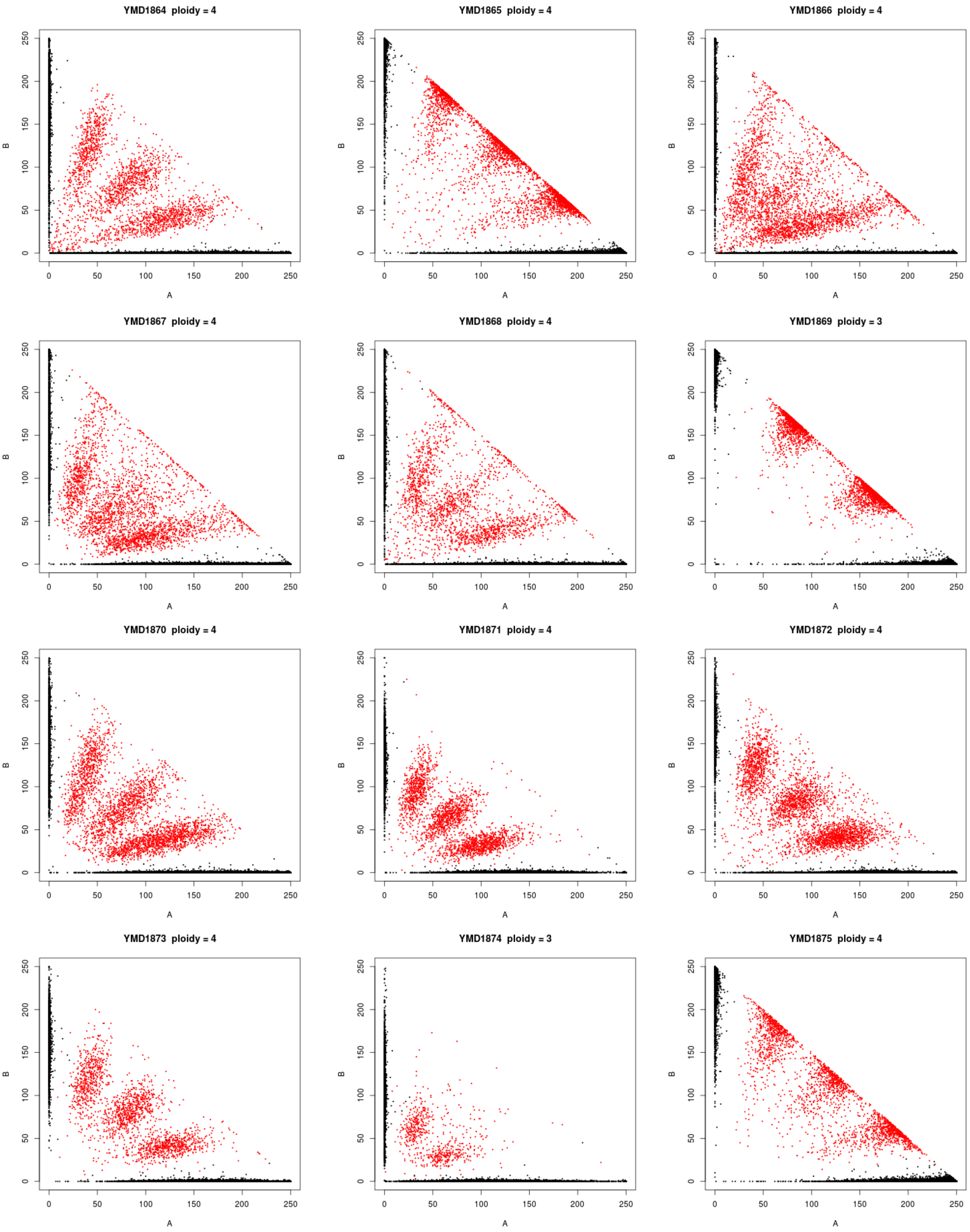
YMD1864 ploidy = 4
YMD1865 ploidy = 4
YMD1866 ploidy = 4
YMD1867 ploidy = 4
YMD1868 ploidy = 4
YMD1869 ploidy = 3
YMD1870 ploidy = 4
YMD1871 ploidy = 4
YMD1872 ploidy = 4
YMD1873 ploidy = 4
YMD1874 ploidy = 3
YMD1875 ploidy = 4
A
B
0
50
100
150
200
250

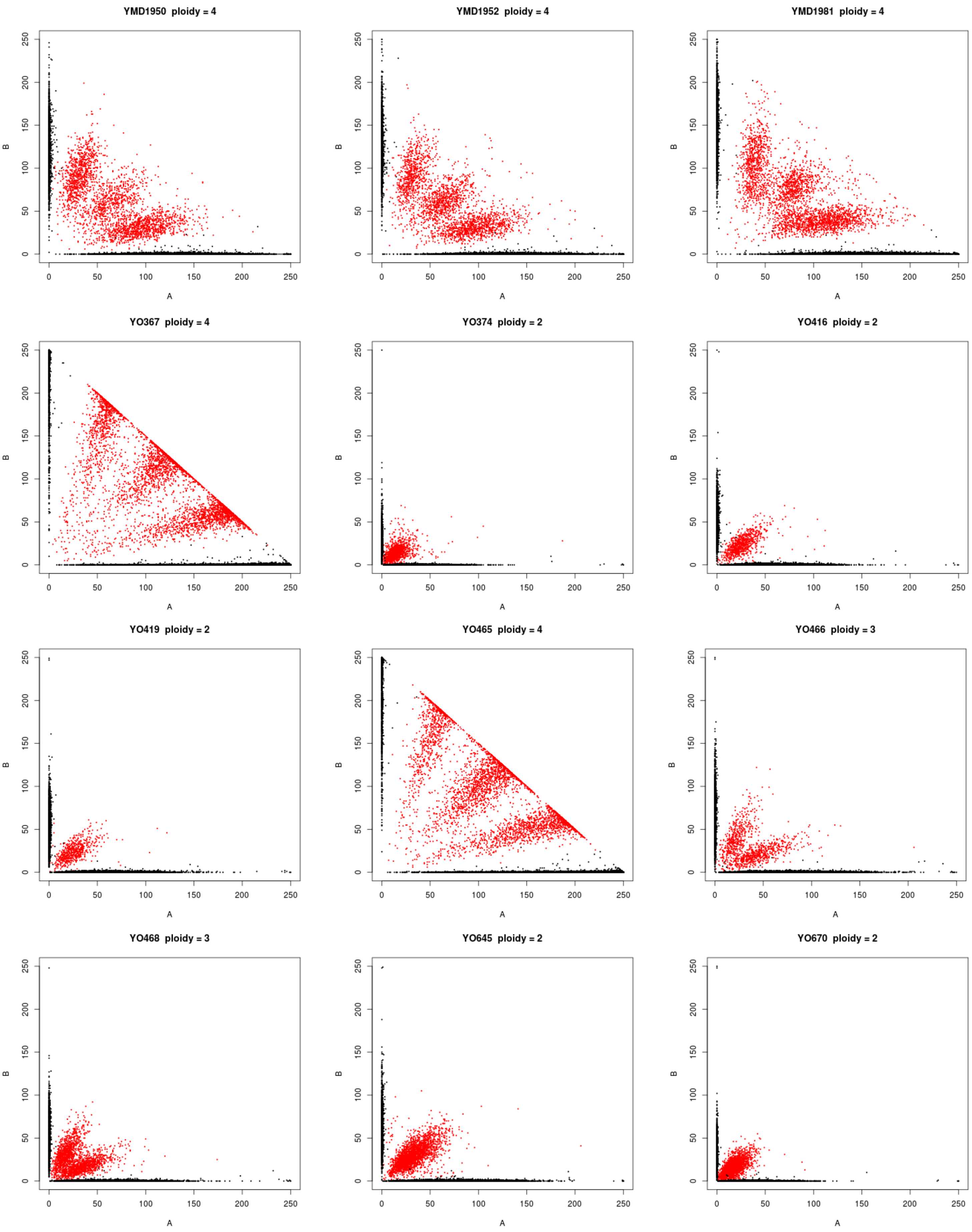

YMD1950 ploidy = 4
YMD1952 ploidy = 4
YMD1981 ploidy = 4
YO367 ploidy = 4
YO374 ploidy = 2
YO416 ploidy = 2
YO419 ploidy = 2
YO465 ploidy = 4
YO466 ploidy = 3
YO468 ploidy = 3
YO645 ploidy = 2
YO670 ploidy = 2
A
B
0
50
100
150
200
250

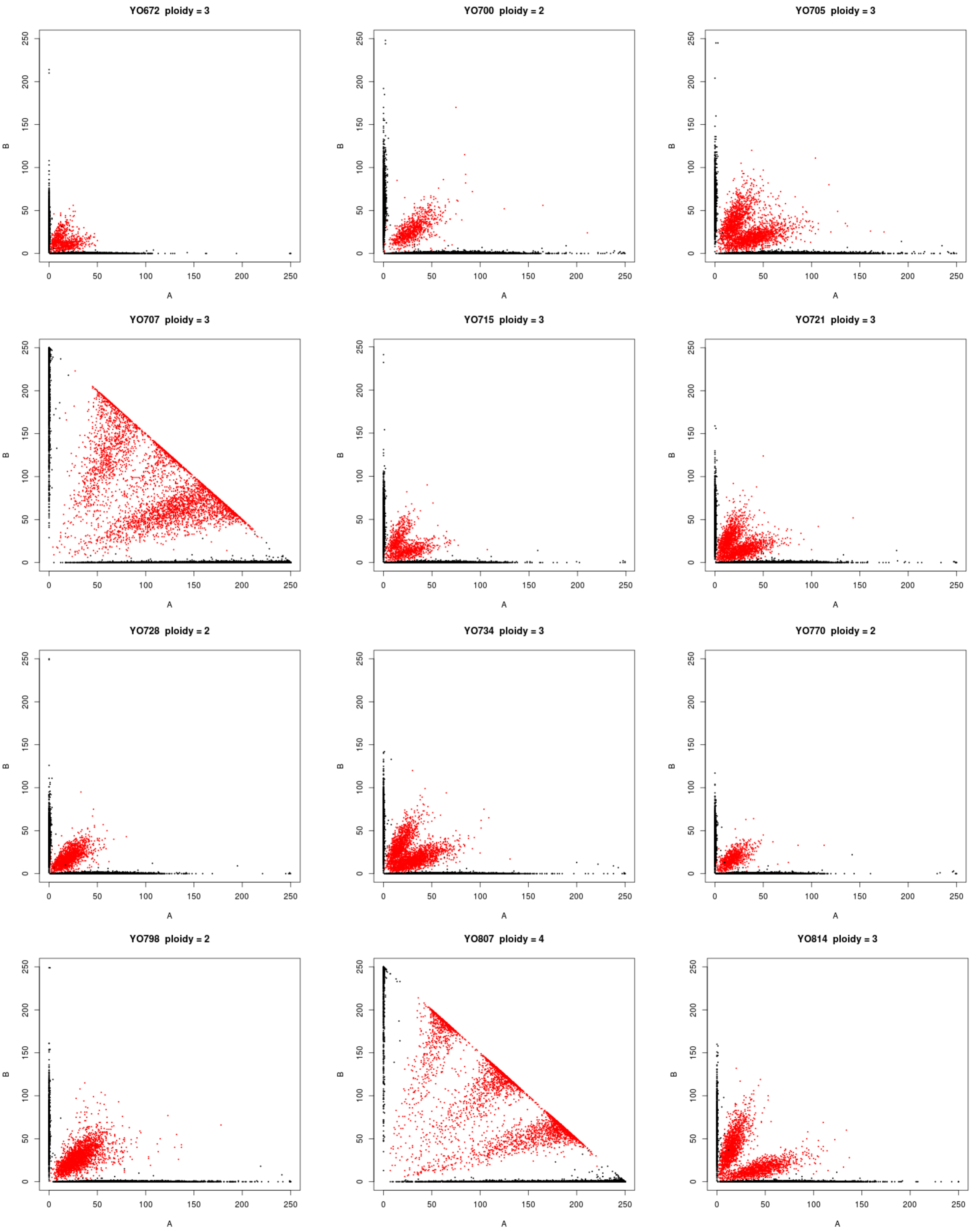
YO672 ploidy = 3
YO700 ploidy = 2
YO705 ploidy = 3
YO707 ploidy = 3
YO715 ploidy = 3
YO721 ploidy = 3
YO728 ploidy = 2
YO734 ploidy = 3
YO770 ploidy = 2
YO798 ploidy = 2
YO807 ploidy = 4
YO814 ploidy = 3
A
B

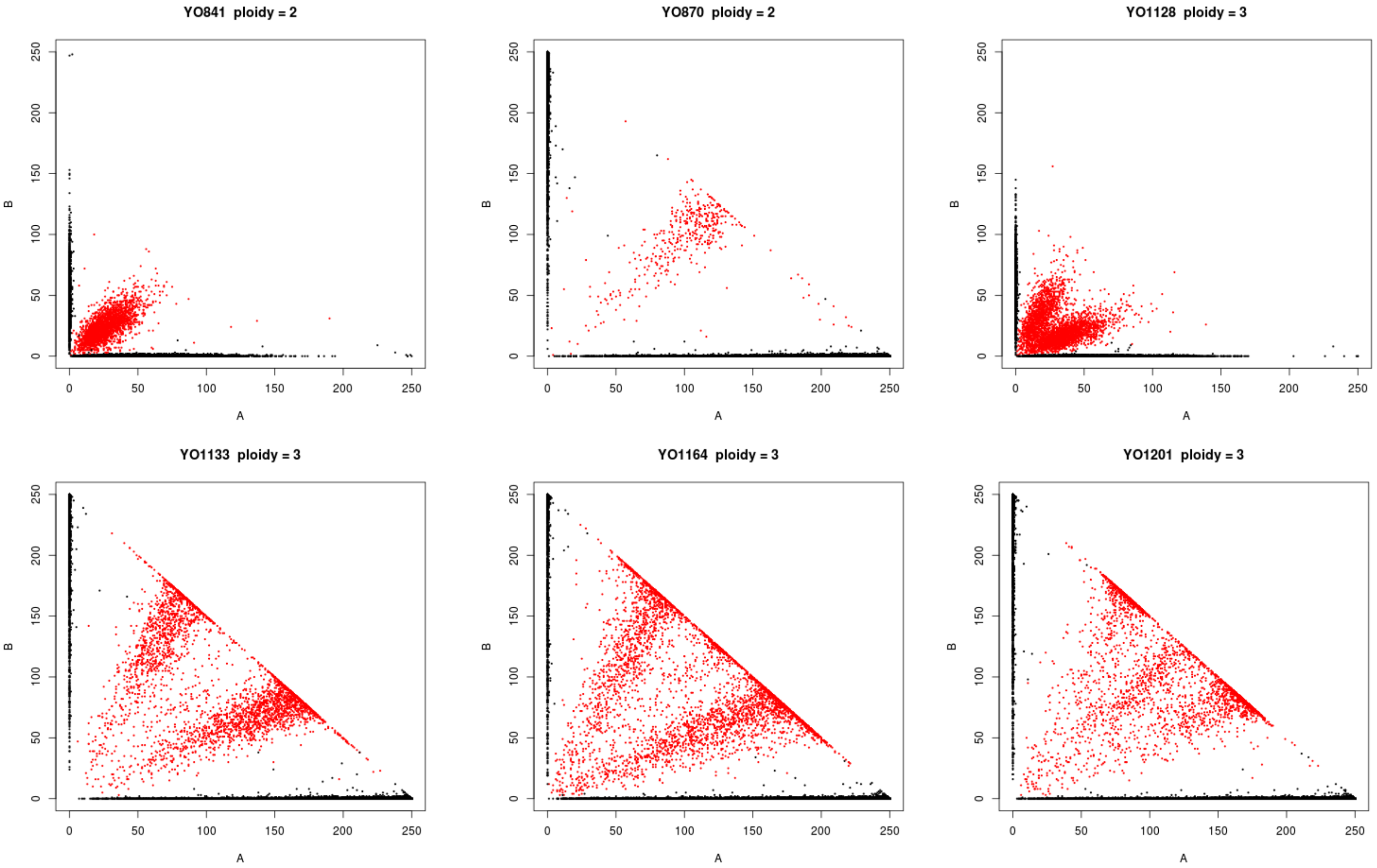
YO841 ploidy = 2
YO870 ploidy = 2
YO1128 ploidy = 3
YO1133 ploidy = 3
YO1164 ploidy = 3
YO1201 ploidy = 3
A
B
0
50
100
150
200
250
