# Supplementary figures and images for: A polyploid admixed origin of beer yeasts derived from European and Asian wine populations
Source: PLoS Biol. 2019 Mar 5;17(3):e3000147. doi: 10.1371/journal.pbio.3000147 (PMC6400334; doi:10.1371/journal.pbio.3000147)

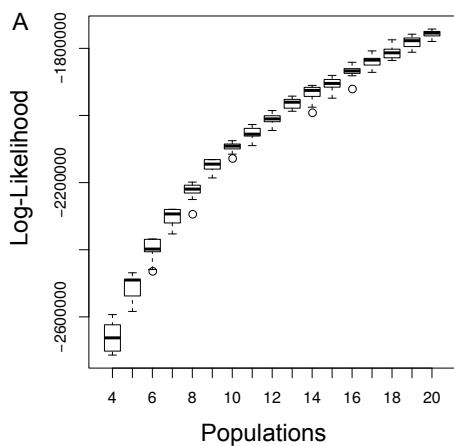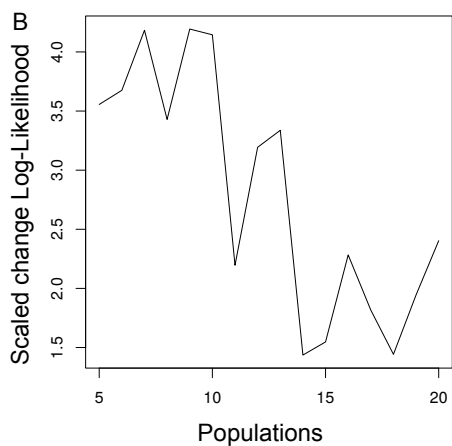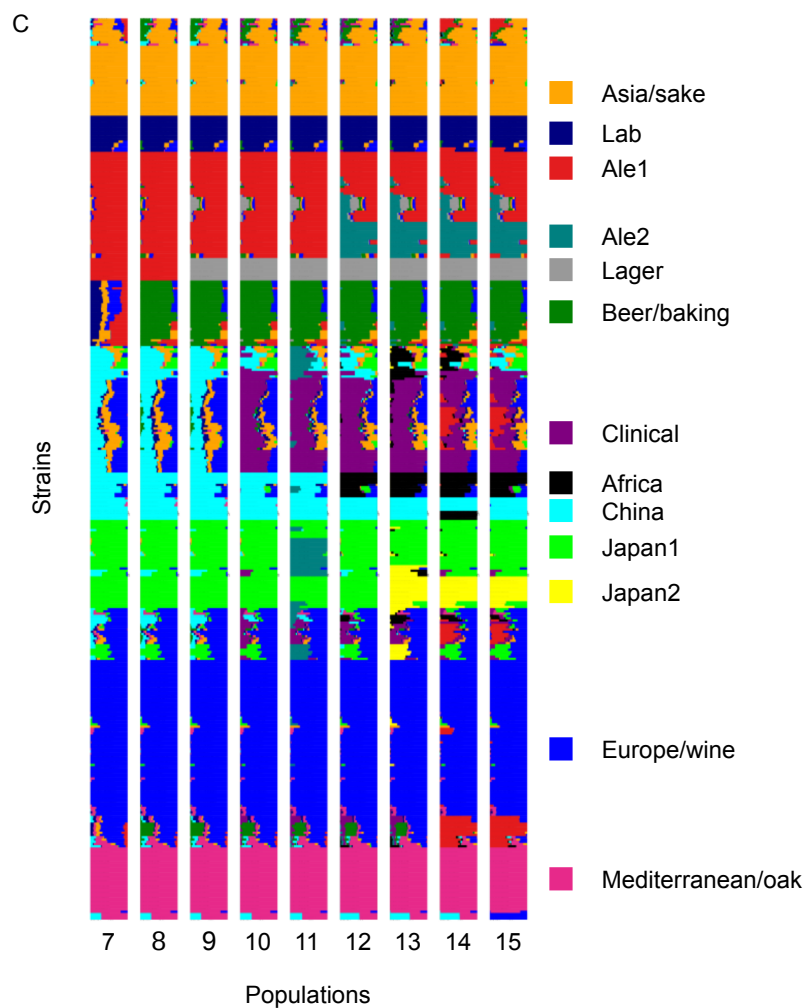

Supplement: S3 Fig — (A) Boxplot of the log-likelihood of 20 independent runs as a function of the number of populations. (B) The scaled improvement in fit, measured by the change in the log-likelihood with increasing population number divided by the standard deviation in the log-likelihood values from 20 independent runs. (C) Population assignments assuming a different number of populations. Each row shows a strain with ancestry to different populations shown by colors and population labels based on similarity to the labels for 13 populations. The data underlying this figure is available from http://doi.org/10.6084/m9.figshare.7550009.v1. (PDF) [file pbio.3000147.s003.pdf]

Hybrid control (YJF1460)

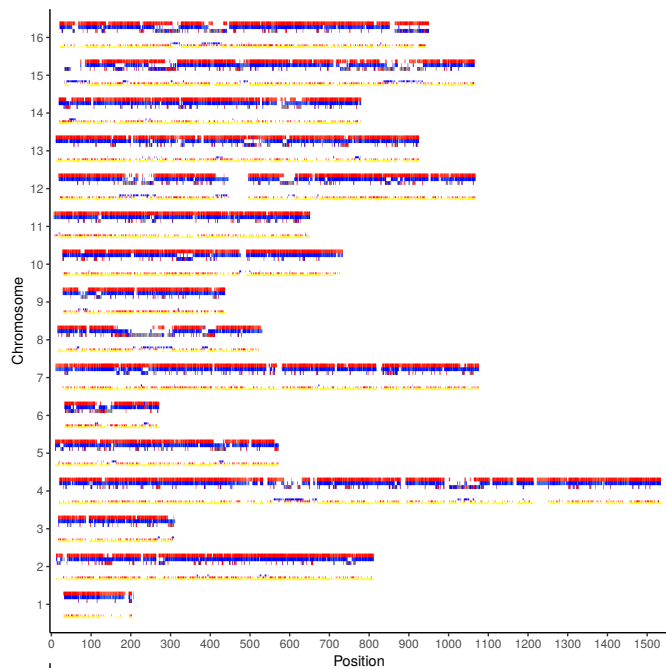

Belgian Ale (T.58)

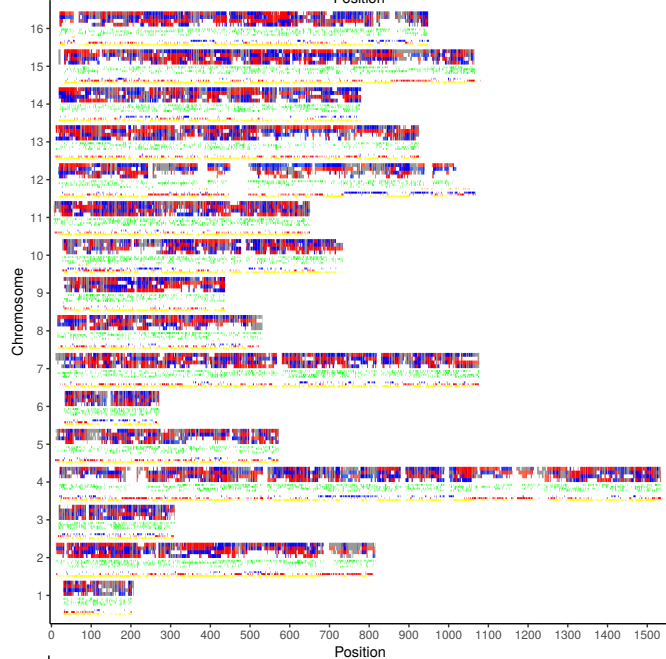

German Ale (A.2565)

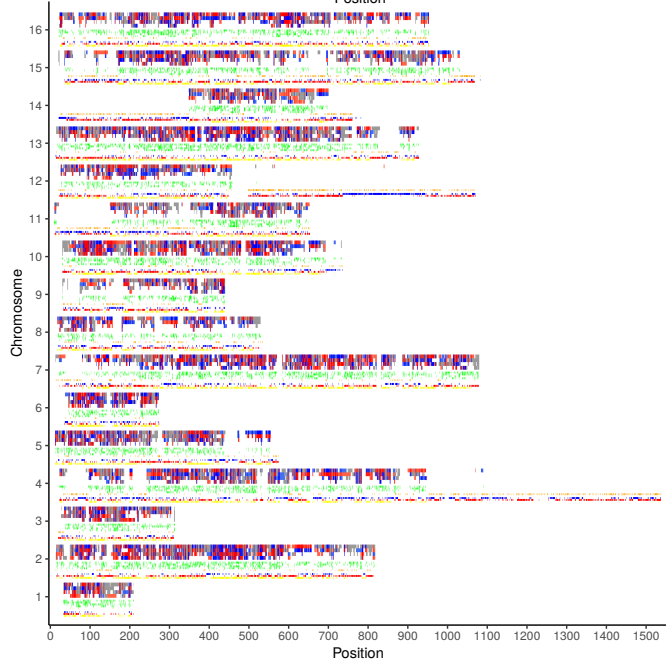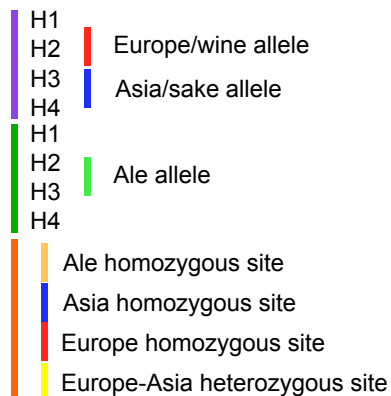

Supplement: S4 Fig — Panels are the same as in Fig 4 and show all 16 chromosomes for two ale strains (T.58 and A.2565) and the control hybrid (YJF1460). European or Asian alleles are shown in red and blue, respectively, and ale alleles in green. The orange panel shows homozygous ale, European and Asian alleles, as well as sites heterozygous for Europe-Asia alleles. The data underlying this figure are available from http://doi.org/10.6084/m9.figshare.7550009.v1. (PDF) [file pbio.3000147.s004.pdf]

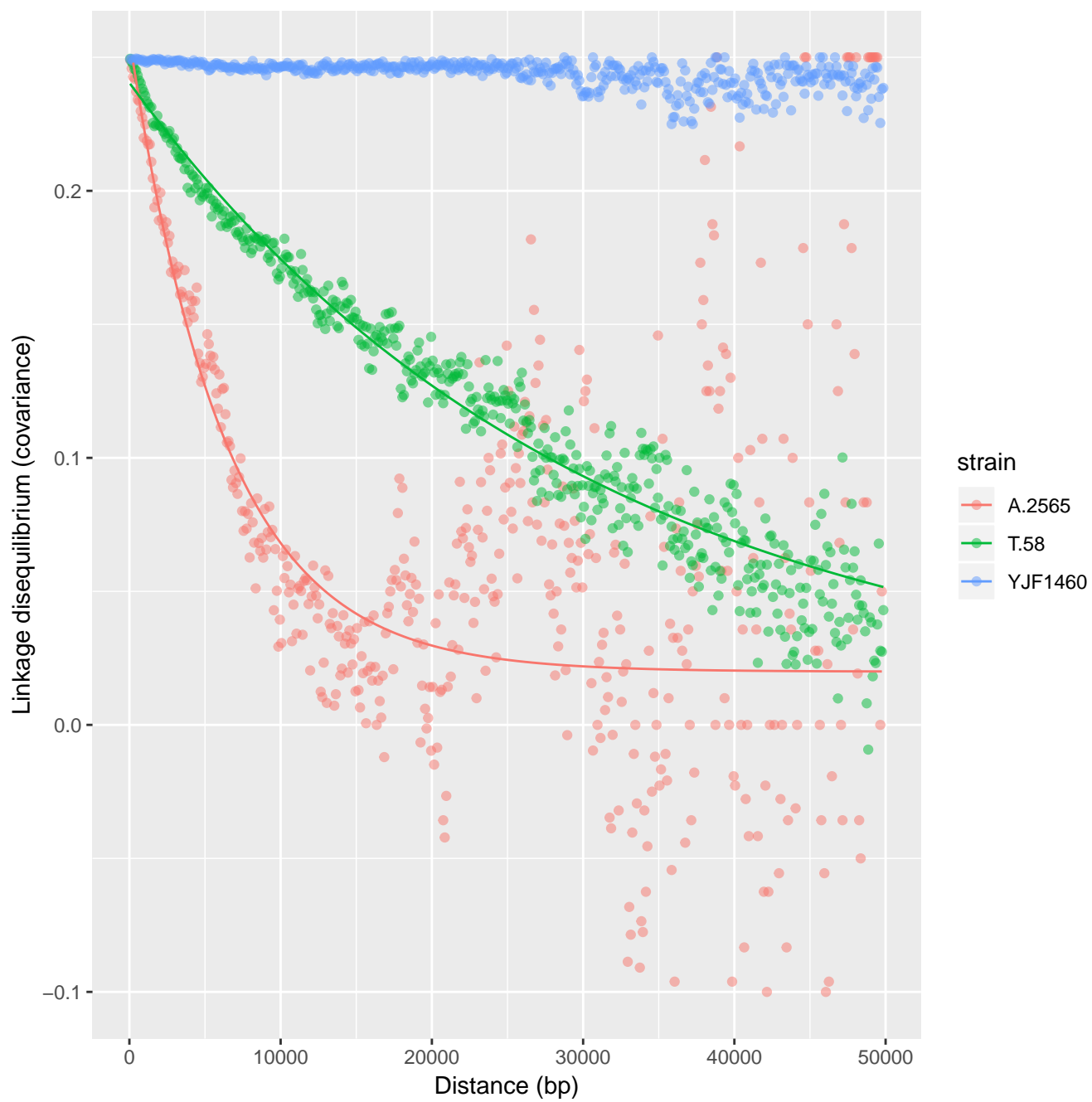

Supplement: S5 Fig — Linkage disequilibrium was measured by the covariance of alleles across sites on the same phased haplotypes for A.2565 (red), T.58 (green), and YJF1460 (blue). Each point shows the average covariance of sites with distances binned into 100 bp increments. The solid lines represent the fit to an exponential decay function. The data underlying this figure are available from http://doi.org/10.6084/m9.figshare.7550009.v1. (PDF) [file pbio.3000147.s005.pdf]
